# DNA as genetic material and as a nutrient in halophilic Archaea

**DOI:** 10.3389/fmicb.2014.00539

**Published:** 2014-10-14

**Authors:** Aharon Oren

**Affiliations:** Department of Plant and Environmental Sciences, The Institute of Life Sciences, The Hebrew University of JerusalemJerusalem, Israel

**Keywords:** *Haloferax*, DNase, phosphate, polyploidy, DNA

Phosphorus is a key element for life, and in many ecosystems phosphate is the limiting nutrient. This is also true for many hypersaline ecosystems. A well-known example is the Dead Sea, where the possibility of massive development of microbial blooms is determined by the excessively high concentrations of magnesium and calcium and phosphate is the limiting inorganic nutrient, not only for the alga *Dunaliella* but also for the halophilic Archaea in blooms that occasionally develop in the lake when conditions become favorable (Oren, 1983). It is therefore not surprising that members of the *Halobacteriaceae* have developed mechanisms to use not only phosphate ions but other phosphorus sources as well. Thus, the square archaeon *Haloquadratum walsbyi* possesses a gene cluster that allows uptake of phosphonates and cleavage of the stable carbon-phosphorus bond (Bolhuis et al., 2006).

DNA contains 10% phosphorus by weight. Many halophilic Archaea have discovered the advantage of DNA as a phosphorus storage polymer: they are commonly polyploid: some species may contain up to 30 genome copies (Breuert et al., 2006; Soppa, 2013; Zerulla and Soppa, 2014). Polyploidy was documented in *Halobacterium salinarum, Haloferax mediterranei*, and *Haloferax volcanii*. Cell sorting of prokaryotes from saltern brines provided further evidence for polyploidy (Zhaxybayeva et al., 2013). When *Hfx. volcanii* cells are starved for phosphorus, the number of genome copies is reduced from ~30 to ~2, allowing for about three additional cell division cycles. The degree of ploidy is thus determined by the level of available nutrients. The number of ribosomes is not decreased, showing that rRNA does not serve as a phosphorus reserve. In non-starved cells the amount of DNA-bound phosphorus is about twice that found in the ribosomes, making DNA more suitable as phosphorus storage material (Zerulla et al., 2014). The stable storage of phosphate was even proposed as a driving force for the emergence of DNA in early evolution before it took over the function of genetic information carrier from RNA (Zerulla and Soppa, 2014; Zerulla et al., 2014).

Another way to obtain phosphorus is by degrading DNA outside the cells. Extracellular DNA is found in all environments, including hypersaline ones (Chimileski et al., 2014). The ability to degrade extracellular DNA is seldom tested during characterization of halophilic Archaea isolates. The proposed minimal standards for description of new taxa of *Halobacteriales* (Oren et al., 1997) include tests for amylase, protease and lipase but not DNAse. There are only few studies where isolates were tested for hydrolytic activity on high-salt DNA-containing agar. Nearly half of 293 archaeal strains isolated from an Iranian salt lake tested positive for DNA degradation; most were *Halorubrum* spp. (Makhdoumi Kakhki et al., 2011). Two out of six representative isolates from Tuz Lake, Turkey and the adjacent salterns—a *Halorubrum* and a *Halobacterium* showed the activity (Birbir et al., 2007), as did two *Halogeometricum*-affiliated isolates from salterns of Tamil Nadu, India (Manikandan et al., 2009). The nature of the enzyme(s) with extracellular DNase activity in *Halobacteriaceae* was never ascertained. An ISI Web of Science search did not yield any papers dedicated to the properties of haloarchaeal DNases.

Mechanisms of degradation of DNA to be used as a nutrient and to be taken up as a source of new genes may be related (Finkel and Kolter, 2001). Horizontal transfer of genetic material is known for a number of halophilic Archaea. In the genus *Haloferax* mating systems are based on direct contact between cells of the same species (Rosenshine et al., 1989) and even of different species, leading to the formation of recombinant hybrids (Naor et al., 2012). Genetic recombination with extensive gene exchange occurs within natural populations of *Halorubrum* (Papke et al., 2004, 2007), but the underlying mechanism is still unknown. Evidence for the transfer of large sections of DNA between phylogenetically disparate members of the *Halobacteriaceae* came from a metagenomic study in Deep Lake, an Antactic hypersaline lake. The four organisms dominating the community and belonging to different genera share contiguous up to 35-kb long regions of ~100% identity (DeMaere et al., 2013).

In a recent “Frontiers in Microbiology” paper, Chimileski et al. (2014) studied extracellular DNA metabolism in *Hfx. volcanii*. They showed that exogenous double-stranded DNA can be used as a nutrient to supply essential phosphorus. Not all sources of DNA are equally suitable, and there is a large degree of specificity involved. DNA of the same species supports growth, but herring sperm DNA or *Escherichia coli* DNA were not effective. However, unmethylated *E. coli* DNA was metabolized. Thus, there is a bias against highly divergent methylated DNA.

The gene *Hvo_1477* was identified as a factor likely involved in DNA processing at the cell surface. It is the first identified archaeal extracellular DNA processing / uptake-related gene. Its deletion created a strain unable to grow on extracellular DNA. *Hvo_1477* homologs are commonly found in genomes of *Halobacteriaceae* and other Archaea. *Hvo_1477* codes for a 327 amino acid lipoprotein attached to the membrane, homologous with known *Bacillus* and *Staphylococcus* nucleases.

Use of DNA as a nutrient may be considered a form of natural DNA uptake or natural competence. However, natural competence requires internalization of intact DNA fragments and the presence of a complex molecular machine for binding and intracellular processing of DNA. Import of high-molecular-weight DNA through membranes of halophilic Archaea is yet to be conclusively demonstrated.

The data presented have important implications for different disciplines including microbial ecology, nutrition, and evolution. They raise some intriguing questions:

- To what extent is extracellular DNA exploited as a phosphorus source in natural communities of *Halobacteriaceae?*
- If the sole purpose of DNA uptake by *Hfx. volcanii* were the sequestering of phosphorus to avoid starvation, why are not all types of DNA taken up with equal efficiency?
- If DNA can be internalized, how does the cell decide whether to degrade it as a source of nutrient or explore the possibility that it may encode useful genetic material?

The study by Chimileski et al. may thus lead to basic new insights in more than one field of microbiology.

## Conflict of interest statement

The author declares that the research was conducted in the absence of any commercial or financial relationships that could be construed as a potential conflict of interest.

## References

[B1] Birbir M. Calli B. Mertoglu B. Elevi Bardavid R. Oren A. Ogmen M. N. (2007). Extremely halophilic Archaea from Tuz Lake, Turkey, and the adjacent Kaldirim and Kayacik salterns. World J. Microbiol. Biotechnol. 23, 309–316 10.1007/s11274-006-9223-4

[B2] Bolhuis H. Palm P. Wende A. Falb M. Rampp M. Rodriguez-Valera F. (2006). The genome of the square archaeon *Haloquadratum walsbyi*: life at the limits of water activity. BMC Genomics 7:169 10.1186/1471-2164-7-169 16820047 PMC1544339

[B3] Breuert S. Allers T. Spohn G. Soppa J. (2006). Regulated polyploidy in halophilic archaea. PLoS ONE 1:e92 10.1371/journal.pone.0000092 17183724 PMC1762399

[B4] Chimileski S. Dolas K. Naor A. Gophna U. Papke R. T. (2014). Extracellular DNA metabolism in *Haloferax volcanii*. Front. Microbiol. 5:57 10.3389/fmicb.2014.00057 24600440 PMC3929857

[B5] DeMaere M. Z. Williams T. J. Allen M. A. Brown M. V. Gibson J. A. E. Rich J. (2013). High level of intergenera gene exchange shapes the evolution of haloarchaea in an isolated Antarctic lake. Proc. Natl. Acad. Sci. U.S.A. 110, 16939–16944 10.1073/pnas/pnas.1307090110 24082106 PMC3801024

[B6] Finkel S. E. Kolter R. (2001). DNA as a nutrient: novel role for bacterial competence gene homologs. J. Bacteriol. 183, 6288–6293 10.1128/JB.183.21.6288-6293.2001 11591672 PMC100116

[B7] Makhdoumi Kakhki A. Amoozegar M. A. Mahmo di Khaledi E. (2011). Diversity of hydrolytic enzymes in haloarchaeal strains isolated from salt lake. Int. J. Environ. Sci. Tech. 8, 705–714 10.1007/BF03326255

[B8] Manikandan M. Kannan V. Pašić L. (2009). Diversity of microorganisms in solar salterns of Tamil Nadu, India. World J. Microbiol. Biotechnol. 25, 1007–1017 10.1007/s11274-009-9980-y

[B9] Naor A. Lapierre P. Mevarech M. Papke R. T. Gophna U. (2012). Low species barriers in halophilic archaea and the formation of recombinant hybrids. Curr. Biol. 22, 1444–1448 10.1016/j.cub.2012.05.056 22748314

[B10] Oren A. (1983). Population dynamics of halobacteria in the Dead Sea water column. Limnol. Oceanogr. 28, 1094–1103 10.4319/lo.1983.28.6.1094

[B11] Oren A. Ventosa A. Grant W. D. (1997). Proposal of minimal standards for description of new taxa in the order *Halobacteriales*. Int. J. Syst. Bacteriol. 47, 233–238 10.1099/00207713-47-1-233

[B12] Papke R. T. Koenig J. E. Rodriguez-Valera F. Doolittle W. F. (2004). Frequent recombination in a saltern population of *Halorubrum*. Science 306, 1928–1929 10.1126/science.1103289 15591201

[B13] Papke R. T. Zhaxybaeeva O. Feil E. J. Sommerfeld K. Muise D. Doolittle W. F. (2007). Searching for species in haloarchaea. Proc. Natl. Acad. Sci. U.S.A. 104, 14092–14097 10.1073/pnas.0706358104 17715057 PMC1955782

[B14] Rosenshine I. Tchelet R. Mevarech M. (1989). The mechanism of DNA transfer in the mating system of an archaebacterium. Science 245, 1387–1389 10.1126/science.2818746 2818746

[B15] Soppa J. (2013). Evolutionary advantages of polyploidy in halophilic Archaea. Biochem. Soc. Trans. 41, 339–343 10.1042/BST/BST212013-150 23356308

[B16] Zerulla K. Chimileski S. Näther D. Gophna U. Papke R. T. Soppa J. (2014). DNA as a phosphate storage polmer and the alternative of polyploidy for growth or survival. PLoS ONE 9:e941819 10.1371/journal.pone.0094819 24733558 PMC3986227

[B17] Zerulla K. Soppa J. (2014). Polyploidy in haloarchaea: advantages for growth and survival. Front. Microbiol. 5:274 10.3389/fmicb.2014.00274 24982654 PMC4056108

[B18] Zhaxybayeva O. Stepanauskas R. Mohan N. R. Papke R. T. (2013). Cell sorting analysis of geographically separated hypersaline environments. Extremophiles 17, 265–275 10.1007/s00792-013-0514-z 23358730

